# Stumbling broke the spleen and unveiled pheochromocytoma, which in turn broke the heart

**DOI:** 10.1007/s12020-019-02169-4

**Published:** 2019-12-27

**Authors:** Shams Y-Hassan, Henrik Falhammar

**Affiliations:** 1 Coronary Artery Disease Area, Heart and Vascular Theme, Karolinska Institutet and Karolinska University Hospital, Stockholm, Sweden; 2 Department of Endocrinology, Metabolism and Diabetes, Karolinska University Hospital, Stockholm, Sweden; 3 Departement of Molecular Medicine and Surgery, Karolinska Institutet, Stockholm, Sweden

**Fig. 1:**
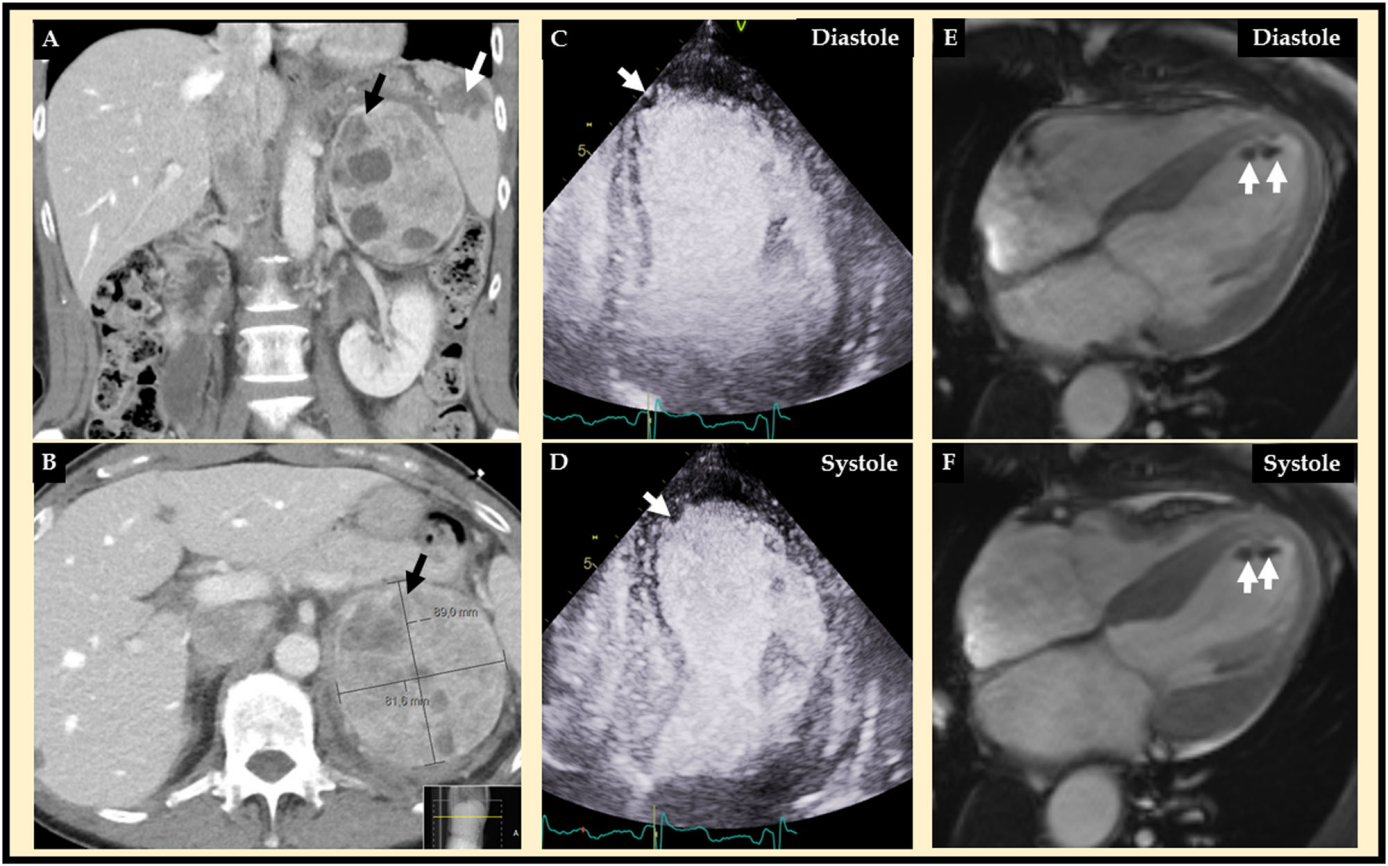
Abdominal computed tomography (CT) (**a** and **b**) revealed a left adrenal tumor, which turned out to be a pheochromocytoma (black arrow), and a rupture of spleen (A white arrow) with retroperitoneal bleeding. Contrast echocardiography (**c** diastole and **d** systole) revealed mid-apical ballooning typical for takotsubo syndrome with an apical filling defect suggestive of left ventricular thrombus (white arrow). Cardiac magnetic resonance (CMR) imaging (**e** diastole and **f** systole) confirmed the mid-apical pattern of takotsubo syndrome and the apical left ventricular thrombus (white arrows)

A 61-year-old woman presented with abdominal and severe chest pain after stumbling over a stone during jogging. The patient had type 2 diabetes mellitus treated with oral medications in addition to a 4–5 years history of episodes of palpitations, anxiety, and high blood pressure. Abdominal computed tomography (CT) revealed a bleeding left heterogenous adrenal tumor measuring 82 × 89 × 115 mm^3^, retroperitoneal bleeding, and ruptured spleen, which was literally broken into two pieces (Fig. 1a, b). Echocardiography revealed mid-apical left ventricular ballooning typical for takotsubo syndrome (TS) (Fig. 1c, d) with suspicion of left ventricular thrombus (LVT, white arrow). Cardiac magnetic resonance imaging confirmed TS with typical mid-apical ballooning pattern of the left ventricle complicated by LVT (Fig. 1e, f white arrows). Native T1 map shows signs of regional myocardial edema in the hypokinetic segments. On late gadolinium enhancement imaging, there was no signs of myocardial infarction. CT coronary angiography showed completely normal left and right coronary arteries. These findings were typical for TS complicated by LVT. The bleeding and rupture of the spleen was treated with embolization of the splenic artery. Marked elevation of both plasma metanephrine and normetanephrine (5.1 and 26 nmol/L, respectively; normal <0.3 and <1.1 nmol/L, respectively) was found. Consequently, stumbling during jogging caused splenic rupture and unmasked the pheochromocytoma, which in turn triggered broken heart syndrome or TS. Because the left ventricular wall motion abnormality in TS is completely reversible, supportive therapy in the form of beta-blockers (bisoprolol), but also alpha-blockers (doxazosin) due the pheochromocytoma, and the treatment of the LVT were initiated. The patient had traumatic abdominal bleeding, which confounded the treatment of LVT with anticoagulation. After careful consideration, the LVT was treated with the low molecular weight heparin dalteparine (5000E mane 7500E nocte sc daily) for 3 weeks followed by enoxaparine (10,000E sc daily) for another 7 weeks. The left ventricular function was normalized, and the left ventricular thrombus was dissolved after a few weeks. The pheochromocytoma was resected successfully with open left adrenalectomy 5 weeks later after treatment with alpha- (doxazosin initiated at 4 mg daily from the suspicion of pheochromocytoma and then slowly up-titrated to 8 mg bd) and beta-blockers (bisoprolol 2.5 mg daily from the diagnosis of TS). Histology revealed a radically extirpated pheochromocytoma. Genetic testing could not demonstrate any familial syndrome. At follow-up 3 months later, the blood pressure was 125/70 without any medications and plasma glucose levels had normalized.

Pheochromocytomas and paragangliomas (PPGLs) are catecholamine-secreting tumors that arise from chromaffin tissue of the sympathetic nervous system [1]. Pheochromocytoma in the current case detected as an adrenal incidentaloma during abdominal imaging after stumbling on stone during jogging. PPGLs is presently known trigger factor for TS. However, the combination of severe abdominal pain, intra-abdominal bleeding, and the surge of plasma catecholamines may have triggered a severe mid-apical pattern of TS in the current case [2]. PPGL-triggered TS is characterized by high cardiovascular complication rates, which may occur in two thirds of patients [2]. TS in our patient was complicated by LVT. LVT has been reported in 1–8% in patients with TS, and thrombo-embolism in 2–14% of patients with TS in general [3]. Thrombo-embolism reported in 7 of 80 of patients (9%) with pheochromocytoma-triggered TS; 6 of 7 patients (86%) had mid-apical pattern of TS as our patient. Normally thrombo-embolism in TS is treated with low molecular weight heparin followed by warfarin for 2–3 months or until the LVT is completely normalized. Our patient was treated for 10 weeks with low molecular weight heparin only, and no warfarin, due to her initial intra-abdominal bleeding.
